# Supplementary material for: Histological, immunohistochemical and transcriptomic characterization of human tracheoesophageal fistulas
Source: PLoS One. 2020 Nov 17;15(11):e0242167. doi: 10.1371/journal.pone.0242167 (PMC7671559; doi:10.1371/journal.pone.0242167)
Supplement: S1 File — (PDF) [file pone.0242167.s001.pdf]

S1 File: Differential expression analysis, clustering and sex differences

S1a: unsupervised hierarchical clustering dendogram

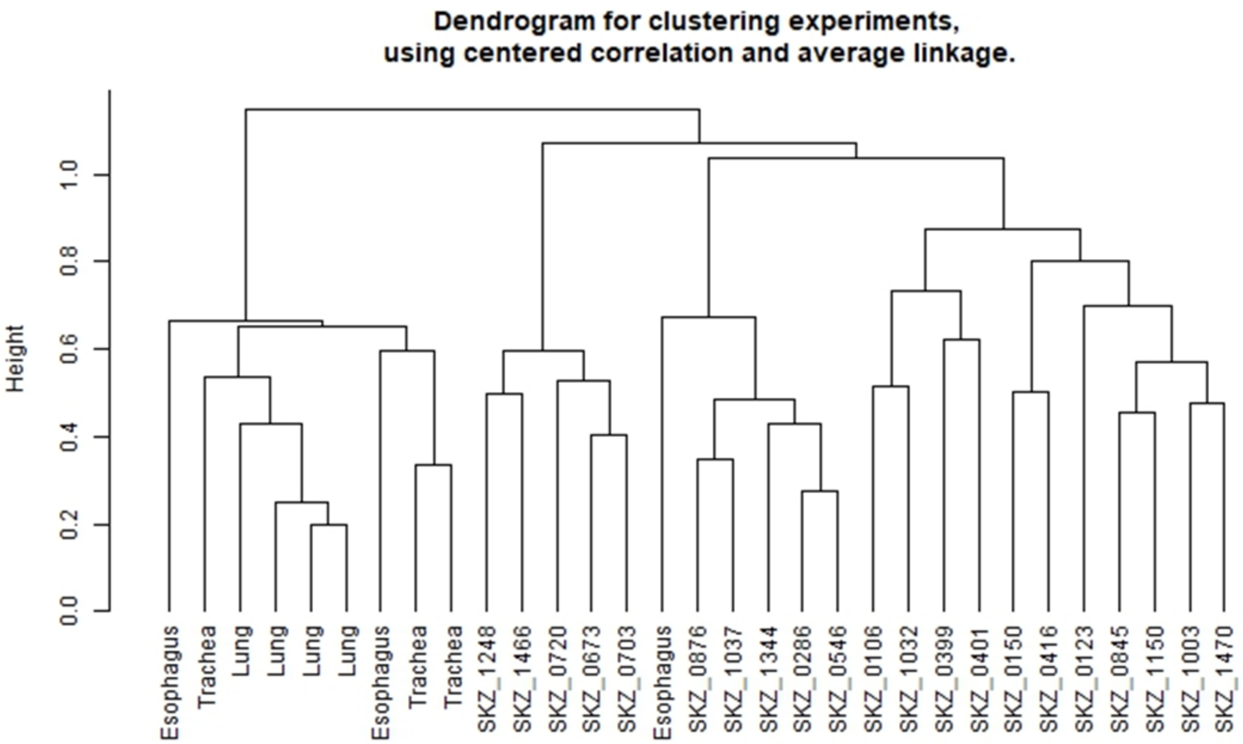

Dendrogram of clustering of all case and control samples F: TEF; T: trachea; E: esophagus; L: lung. For clarity, TEF, E, T and L samples are of different individuals We compared the transcriptomes of 21 TEFs to 3 esophageal, 3 tracheal and 4 lung samples. Unsupervised hierarchical clustering of all samples showed that TEF differ from the control samples (lung, trachea and esophagus) based on their whole genome transcription profiles (supplementary data 1b). All TEFs clearly clustered together separately from lung and tracheal tissue. Whereas the lung tissue samples also clustered separately, esophageal and tracheal tissue showed more mixed patterns.

S1b: number of differentially expressed genes (DEG) per tissue type

	DEG (n)
**TEF vs Esophagus**	1381↑, 1211↓
**TEF vs Lung**	2986↑, 3141↓
**TEF vs Trachea**	2048↑, 1920↓
**TEF vs Esophagus and Trachea**	1397↑, 1259↓

	Esophagus	Trachea	Lung
**Esophagus**	0	425	2080
**Trachea**	425	0	2177
**Lung**	2080	2177	0

Top panel: depicted are the number of differentially expressed genes (DEG) between tissue types and TEF in individual comparisons using the random variance t-test (RV t-test) at 0.05 significance and number of up- and down-regulated genes between tissue types and TEF in individual comparisons using the random variance t-test (RV t-test) at 0.05 significance . Bottom panel: DEG between control tissue types in individual comparisons using the random variance t-test (RV t-test) at 0.05 significance. We compared the expression patterns of these control tissues individually to the TEF. TEF mostly resembled esophagus based on the low number of differentially expressed genes.

S1c: Top 50 differential expressed genes when comparing esophagus and TEF

Rank	Parametric P-value	FDR	TEF	E	T	L	Foldchange	Symbol	Name	EntrezID
**1**	< 1e-07	< 1e-07	1719.36	101.81	141.73	80.43	-16.888	KCNMB1	potassium calcium-activated channel subfamily M regulatory beta subunit 1	3779
**2**	< 1e-07	< 1e-07	5323.59	267.48	178.14	144.79	-19.903	SYNM	synemin	23336
**3**	< 1e-07	< 1e-07	698.96	63.61	91.31	81.83	-10.988	FAM83D	family with sequence similarity 83 member D	81610
**4**	< 1e-07	< 1e-07	6108.81	150.95	233.83	124.38	-40.469	CNN1	calponin 1	1264
**5**	< 1e-07	< 1e-07	3708.21	120.57	118.79	40.65	-30.756	SYNPO2	synaptopodin 2	171024
**6**	< 1e-07	< 1e-07	1017.83	45.01	165.66	73.64	-22.613	PLN	phospholamban	5350
**7**	< 1e-07	< 1e-07	768.68	29.47	35.52	21.29	-26.083	MBNL1-AS1	MBNL1 antisense RNA 1	401093
**8**	< 1e-07	< 1e-07	5547.58	1154.23	878.38	972.03	-4.806	CSRP1	cysteine and glycine rich protein 1	1465
**9**	< 1e-07	< 1e-07	1616.07	126.32	157.95	160.03	-12.793	SMTN	smoothelin	6525
**10**	< 1e-07	< 1e-07	1156.59	72.24	58.12	27.13	-16.01	LMOD1	leiomodin 1	25802
**11**	< 1e-07	< 1e-07	5626.32	702.63	568.76	550.08	-8.008	MYL9	myosin light chain 9	10398
**12**	< 1e-07	< 1e-07	357.31	17.47	14.6	15.86	-20.453	LINC00702	long intergenic non-protein coding RNA 702	100652988
**13**	< 1e-07	< 1e-07	514.68	43.25	55.79	167.72	-11.9	MYOCD	myocardin	93649
**14**	< 1e-07	< 1e-07	1729	281.13	350.66	85.65	-6.15	SELENOM	selenoprotein M	140606
**15**	< 1e-07	< 1e-07	887.23	65.85	84.87	48.43	-13.474	TAGLN	transgelin	6876
**16**	< 1e-07	< 1e-07	2475.66	342.01	373.05	425.47	-7.239	FLNA	filamin A	2316
**17**	< 1e-07	< 1e-07	253.97	23.11	72.73	20.18	-10.99	CPXM2	carboxypeptidase X, M14 family member 2	119587
**18**	< 1e-07	< 1e-07	124.28	11.81	11.54	17.39	-10.523	PLA2G5	phospholipase A2 group V	5322
**19**	< 1e-07	< 1e-07	202.63	20.55	45.69	31.31	-9.86	NFASC	neurofascin	23114
**20**	< 1e-07	< 1e-07	262.22	1000.43	622.57	1356.16	3.815	CADM1	cell adhesion molecule 1	23705
**21**	< 1e-07	3.48e-05	207.2	16.95	14.03	31.52	-12.224	MYLK	myosin light chain kinase	4638
**22**	< 1e-07	3.48e-05	2828.05	253.35	491.28	261.21	-11.163	SORBS1	sorbin and SH3 domain containing 1	10580
**23**	< 1e-07	3.48e-05	18.55	98.44	25.16	24.75	5.307	LRRC2	leucine rich repeat containing 2	79442
**24**	< 1e-07	3.48e-05	450.36	55.93	38.59	67.45	-8.052	FAM46B	family with sequence similarity 46 member B	115572
**25**	0.0000001	3.48e-05	631.88	126.73	82.48	50.88	-4.986	SYNC	syncoilin, intermediate filament protein	81493
**26**	0.0000001	3.48e-05	208.58	105.33	108.46	127.43	-1.98	CLP1	cleavage and polyadenylation factor I subunit 1	10978
**27**	0.0000001	3.48e-05	8613.55	994.58	979.01	557.9	-8.66	TPM2	tropomyosin 2	7169

**28**	0.0000002	6.07e-05	529.34	156.78	208.6	404.77	-3.376	RASL12	RAS like family 12	51285
**29**	0.0000002	6.07e-05	158.8	19.85	20.87	47.09	-8	MICALL1	MICAL like 1	85377
**30**	0.0000002	6.07e-05	558.4	3728.08	4127.59	3118.3	6.676	MEST	mesoderm specific transcript	4232
**31**	0.0000002	6.07e-05	858.81	216.05	191.18	113.38	-3.975	C3orf70	chromosome 3 open reading frame 70	285382
**32**	0.0000003	8.06e-05	149.18	78.26	62.82	71.54	-1.906	C16orf91	chromosome 16 open reading frame 91	283951
**33**	0.0000003	8.06e-05	408.42	84.83	71.91	22.92	-4.815	AHNAK2	AHNAK nucleoprotein 2	113146
**34**	0.0000003	8.06e-05	3623.3	136.06	71.04	51.99	-26.63	DES	desmin	1674
**35**	0.0000003	8.06e-05	716.45	93.23	98.18	83.78	-7.685	GPM6A	glycoprotein M6A	2823
**36**	0.0000004	8.75e-05	12547.21	975.48	1270.12	604.38	-12.863	ACTG2	actin, gamma 2, smooth muscle, enteric	72
**37**	0.0000004	8.75e-05	476.68	1086.39	1291.98	1043.16	2.279	SH3BP5	SH3 domain binding protein 5	9467
**38**	0.0000004	8.75e-05	924.11	178.55	151.11	75.2	-5.176	MRGPRF	MAS related GPR family member F	116535
**39**	0.0000004	8.75e-05	125.47	34.87	32.36	20.92	-3.598	FAXC	failed axon connections homolog	84553
**40**	0.0000004	8.75e-05	348.59	38.63	60.41	14.91	-9.024	PYGB	glycogen phosphorylase B	5834
**41**	0.0000004	8.75e-05	1452.6	163.56	248.5	185.19	-8.881	PPP1R12B	protein phosphatase 1 regulatory subunit 12B	4660
**42**	0.0000004	8.75e-05	332.93	79.03	59.88	108.89	-4.213	PARVA	parvin alpha	55742
**43**	0.0000004	8.75e-05	1822.77	251.74	343.03	352.8	-7.241	PGM5	phosphoglucomutase 5	5239
**44**	0.0000005	1e-04	290.29	81.25	100.49	78.14	-3.573	C8orf88	chromosome 8 open reading frame 88	100127983
**45**	0.0000005	1e-04	295.52	36.84	86.49	56.64	-8.022	NRP2	neuropilin 2	8828
**46**	0.0000005	1e-04	326.11	1013.04	992.06	906.88	3.106	LDB2	LIM domain binding 2	9079
**47**	0.0000005	0.000106	1595.55	278.75	275.5	450.98	-5.724	S1PR3	sphingosine-1-phosphate receptor 3	1903
**48**	0.0000006	0.000106	521.88	129.67	234.3	83.79	-4.025	AK4	adenylate kinase 4	205
**49**	0.0000006	0.000106	100.58	315.45	613.57	593.03	3.136	PTPRD	protein tyrosine phosphatase, receptor type D	5789
**50**	0.0000006	0.000106	97.2	302.41	176.24	444.27	3.11	CACHD1	cache domain containing 1	57685

Depicted are the geometric measures of intensity (GMI) for the groups: TEF; tracheoesophageal fistula, E; esophagus, T; trachea, L; Lung. Foldchange and statistics represent the comparison of esophagus and tracheoesophageal fistula. Genes are ranked on their p-value of the univariate test. FDR corrected p-values are depicted in the adjacent column. Type of univariate test used: Two-sample T-test. Permutation p-values for significant genes were computed based on 10000 random permutations. Nominal significance level of each univariate test: 0.05. Confidence level of false discovery rate assessment: 80 %, Maximum allowed proportion of false-positive genes: 0.01. Number of significant genes by controlling the proportion of false positive genes: 250.

S1d: Top 50 differential expressed genes when comparing trachea and TEF

Rank	Parametric P-value	FDR	TEF	E	T	L	Foldchange	Symbol	Name	EntrezID
**1**	< 1e-07	< 1e-07	12547.21	975.48	1270.12	604.38	-9.879	ACTG2	actin, gamma 2, smooth muscle, enteric	72
**2**	< 1e-07	< 1e-07	6108.81	150.95	233.83	124.38	-26.125	CNN1	calponin 1	1264
**3**	< 1e-07	< 1e-07	193.24	35.64	10	12.34	-19.324	ASB2	ankyrin repeat and SOCS box containing 2	51676
**4**	< 1e-07	< 1e-07	5323.59	267.48	178.14	144.79	-29.884	SYNM	synemin	23336
**5**	< 1e-07	< 1e-07	3623.3	136.06	71.04	51.99	-51.004	DES	desmin	1674
**6**	< 1e-07	< 1e-07	8613.55	994.58	979.01	557.9	-8.798	TPM2	tropomyosin 2	7169
**7**	< 1e-07	< 1e-07	3708.21	120.57	118.79	40.65	-31.217	SYNPO2	synaptopodin 2	171024
**8**	< 1e-07	< 1e-07	13.06	283.76	284.25	54.38	21.765	SLC26A7	solute carrier family 26 member 7	115111
**9**	< 1e-07	< 1e-07	1719.36	101.81	141.73	80.43	-12.131	KCNMB1	potassium calcium-activated channel subfamily M regulatory beta subunit 1	3779
**10**	< 1e-07	< 1e-07	751.91	181.48	57.02	83.98	-13.187	HACD1	3-hydroxyacyl-CoA dehydratase 1	9200
**11**	< 1e-07	< 1e-07	5626.32	702.63	568.76	550.08	-9.892	MYL9	myosin light chain 9	10398
**12**	< 1e-07	< 1e-07	13.49	61.21	140.8	201.2	10.437	SEMA3D	semaphorin 3D	223117
**13**	< 1e-07	< 1e-07	1156.59	72.24	58.12	27.13	-19.9	LMOD1	leiomodin 1	25802
**14**	< 1e-07	< 1e-07	5547.58	1154.23	878.38	972.03	-6.316	CSRP1	cysteine and glycine rich protein 1	1465
**15**	< 1e-07	< 1e-07	386.1	80.47	27.23	69.85	-14.179	EPPK1	epiplakin 1	83481
**16**	< 1e-07	< 1e-07	2901.55	293.84	298.34	129.73	-9.726	PDLIM3	PDZ and LIM domain 3	27295
**17**	< 1e-07	< 1e-07	568.52	119.88	58.93	55.03	-9.647	NACC2	NACC family member 2	138151
**18**	< 1e-07	< 1e-07	357.31	17.47	14.6	15.86	-24.473	LINC00702	long intergenic non-protein coding RNA 702	100652988
**19**	< 1e-07	< 1e-07	698.96	63.61	91.31	81.83	-7.655	FAM83D	family with sequence similarity 83 member D	81610
**20**	< 1e-07	< 1e-07	631.88	126.73	82.48	50.88	-7.661	SYNC	syncoilin, intermediate filament protein	81493
**21**	< 1e-07	< 1e-07	100.58	315.45	613.57	593.03	6.1	PTPRD	protein tyrosine phosphatase, receptor type D	5789
**22**	< 1e-07	< 1e-07	36.84	218.38	804.39	313.48	21.835	COL6A6	collagen type VI alpha 6 chain	131873
**23**	< 1e-07	< 1e-07	768.68	29.47	35.52	21.29	-21.641	MBNL1-[illegible]	MBNL1 antisense RNA 1	401093
**24**	< 1e-07	< 1e-07	478.4	147.08	65.21	48.51	-7.336	MSRB1	methionine sulfoxide reductase B1	51734
**25**	< 1e-07	< 1e-07	4763.6	697.2	422.4	464.91	-11.277	TPM1	tropomyosin 1	7168
**26**	< 1e-07	< 1e-07	450.36	55.93	38.59	67.45	-11.67	FAM46B	family with sequence similarity 46 member B	115572
**27**	< 1e-07	< 1e-07	514.68	43.25	55.79	167.72	-9.225	MYOCD	myocardin	93649

**28**	< 1e-07	< 1e-07	43.66	94.53	191.1	222.92	4.377	ADGRL3	adhesion G protein-coupled receptor L3	23284
**29**	< 1e-07	< 1e-07	42.12	92.72	234.53	60.01	5.568	LMF1	lipase maturation factor 1	64788
**30**	< 1e-07	< 1e-07	207.2	16.95	14.03	31.52	-14.768	MYLK	myosin light chain kinase	4638
**31**	< 1e-07	< 1e-07	1616.07	126.32	157.95	160.03	-10.232	SMTN	smoothelin	6525
**32**	< 1e-07	< 1e-07	179.4	359.1	1099.64	1252.98	6.13	COL21A1	collagen type XXI alpha 1 chain	81578
**33**	< 1e-07	< 1e-07	364.2	89.13	62.46	80.67	-5.831	NTN1	netrin 1	9423
**34**	< 1e-07	< 1e-07	1503.4	279.27	237.63	258.57	-6.327	ACTN1	actinin alpha 1	87
**35**	< 1e-07	< 1e-07	924.11	178.55	151.11	75.2	-6.115	MRGPRF	MAS related GPR family member F	116535
**36**	< 1e-07	< 1e-07	476.68	1086.39	1291.98	1043.16	2.71	SH3BP5	SH3 domain binding protein 5	9467
**37**	< 1e-07	< 1e-07	688.16	98.16	82.2	134	-8.372	PPP1R14A	protein phosphatase 1 regulatory inhibitor subunit 14A	94274
**38**	< 1e-07	< 1e-07	316.42	961.6	1234.35	282.03	3.901	THBS2	thrombospondin 2	7058
**39**	< 1e-07	< 1e-07	2475.66	342.01	373.05	425.47	-6.636	FLNA	filamin A	2316
**40**	< 1e-07	< 1e-07	223.97	41.93	19.61	18.37	-11.421	KCND3	potassium voltage-gated channel subfamily D member 3	3752
**41**	< 1e-07	< 1e-07	224.6	25.21	19.76	17.14	-11.366	ITGB1BP2	integrin subunit beta 1 binding protein 2	26548
**42**	< 1e-07	< 1e-07	124.28	11.81	11.54	17.39	-10.769	PLA2G5	phospholipase A2 group V	5322
**43**	< 1e-07	< 1e-07	289.82	47.04	42.65	39.77	-6.795	CCDC69	coiled-coil domain containing 69	26112
**44**	< 1e-07	< 1e-07	558.4	3728.08	4127.59	3118.3	7.392	MEST	mesoderm specific transcript	4232
**45**	< 1e-07	< 1e-07	651.34	231.94	106.34	120.29	-6.125	SLC25A4	solute carrier family 25 member 4	291
**46**	< 1e-07	< 1e-07	167.51	1075.77	1601.23	1250.4	9.559	PEG10	paternally expressed 10	23089
**47**	< 1e-07	< 1e-07	332.93	79.03	59.88	108.89	-5.56	PARVA	parvin alpha	55742
**48**	< 1e-07	< 1e-07	186.54	102.59	76.23	85.12	-2.447	HCCS	holocytochrome c synthase	3052
**49**	< 1e-07	< 1e-07	887.23	65.85	84.87	48.43	-10.454	TAGLN	transgelin	6876
**50**	< 1e-07	< 1e-07	408.42	84.83	71.91	22.92	-5.68	AHNAK2	AHNAK nucleoprotein 2	113146

Depicted are the geometric measures of intensity (GMI) for the groups: TEF; tracheoesophageal fistula, E; esophagus, T; trachea, L; Lung. Foldchange and statistics represent the comparison of trachea and tracheoesophageal fistula. Genes are ranked on their p-value of the univariate test. FDR corrected p-values are depicted in the adjacent column. Type of univariate test used: Two-sample T-test. Permutation p-values for significant genes were computed based on 10000 random permutations. Nominal significance level of each univariate test: 0.05. Confidence level of false discovery rate assessment: 80 %, Maximum allowed proportion of false-positive genes: 0.01. Number of significant genes by controlling the proportion of false positive genes: 740.

S1e: Sex differences

	Parametric p-value	FDR	Permutation p-value	Geom mean of intensities in F (8 samples)	Geom mean of intensities in M (13 samples).	Fold-change	ProbeSet	Symbol	Name	DefinedGenelist
**1**	< 1e-07	< 1e-07	1e-04	12.79	118.2	0.11	236694_at	TXLNGY	taxilin gamma pseudogene, Y-linked	
**2**	< 1e-07	< 1e-07	1e-04	19.35	623.68	0.031	205000_at	DDX3Y	DEAD-box helicase 3, Y-linked	RIG-I-like receptor signaling pathway
**3**	< 1e-07	< 1e-07	1e-04	15.69	486.77	0.032	204409_s_at	EIF1AY	eukaryotic translation initiation factor 1A, Y-linked	RNA transport
**4**	< 1e-07	< 1e-07	< 1e-07	12.46	127.46	0.098	230760_at	ZFY	zinc finger protein, Y-linked	
**5**	< 1e-07	< 1e-07	1e-04	14.14	144.23	0.098	228492_at	USP9Y	ubiquitin specific peptidase 9, Y-linked	
**6**	< 1e-07	< 1e-07	1e-04	21.93	312.35	0.07	206700_s_at	KDM5D	lysine demethylase 5D	
**7**	< 1e-07	< 1e-07	< 1e-07	15.12	62.61	0.24	214983_at	TTTY15	testis-specific transcript, Y-linked 15 (non-protein coding)	
**8**	< 1e-07	< 1e-07	1e-04	14.13	70.11	0.2	207703_at	NLGN4Y	neuroligin 4, Y-linked	
**9**	< 1e-07	< 1e-07	1e-04	58.56	2864.18	0.02	201909_at	RPS4Y1	ribosomal protein S4, Y-linked 1	Ribosome
**10**	3e-07	0.000282	< 1e-07	11.65	37.92	0.31	211149_at	UTY	ubiquitously transcribed tetratricopeptide repeat containing, Y-linked	

Type of univariate test used: Two-sample T-test with random variance model. The first 10 genes are significant using a False discovery rate of 0.1 with the fold change 1.5. Permutation p-values for significant genes were computed based on 10000 random permutations. Nominal significance level of each univariate test: 0.05
