# Supplementary material for: Histological, immunohistochemical and transcriptomic characterization of human tracheoesophageal fistulas
Source: PLoS One. 2020 Nov 17;15(11):e0242167. doi: 10.1371/journal.pone.0242167 (PMC7671559; doi:10.1371/journal.pone.0242167)
Supplement: S2 File — (PDF) [file pone.0242167.s002.pdf]

S2 File: TEF defining genes

2a Mostly present in TEF, not in Esophagus or Trachea

Rank	Parametric p-value	FDR	Permutation p-value	Geom mean of intensities in Controls (E + T)	Geom mean of intensities in TEF	Fold-change	Symbol	Name	EntrezID	Accession	UGCluster	Times present in TEF
**1**	< 1e-07	< 1e-07	< 1e-07	11.67	124.28	0.094	PLA2G5	phospholipase A2 group V	5322	AL158172	Hs.319438	17
**2**	< 1e-07	< 1e-07	< 1e-07	13.03	150.74	0.086	PTGS1	prostaglandin-endoperoxide synthase 1	5742	NM_000962	Hs.201978	21
**3**	< 1e-07	< 1e-07	< 1e-07	30.62	127.72	0.24	PDLIM3	PDZ and LIM domain 3	27295	W86910	Hs.701364	20
**4**	< 1e-07	< 1e-07	< 1e-07	12.13	107.7	0.11	GABRA5	gamma-aminobutyric acid type A receptor alpha5 subunit	2558	NM_000810	Hs.24969	20
**5**	< 1e-07	< 1e-07	< 1e-07	39.94	342.74	0.12	RBPMS2	RNA binding protein, mRNA processing factor 2	348093	BE348466	Hs.436518	21
**6**	< 1e-07	< 1e-07	< 1e-07	97.93	478.4	0.2	MSRB1	methionine sulfoxide reductase B1	51734	NM_016332	Hs.655346	17
**7**	< 1e-07	< 1e-07	< 1e-07	21.07	82.77	0.25	MYL9	myosin light chain 9	10398	AA864758	Hs.504687	18
**8**	< 1e-07	< 1e-07	< 1e-07	21.07	114.04	0.18	KCNMA1	potassium calcium-activated channel subfamily M alpha 1	3778	AI129381	Hs.144795	18
**9**	< 1e-07	< 1e-07	< 1e-07	19.67	123.34	0.16	DTNA	dystrobrevin alpha	1837	NM_001390	Hs.643454	19
**10**	< 1e-07	< 1e-07	< 1e-07	22.23	145.59	0.15	FBXO32	F-box protein 32	114907	AU155376	Hs.403933	18
**11**	< 1e-07	< 1e-07	< 1e-07	56.45	295.52	0.19	NRP2	neuropilin 2	8828	NM_018534	Hs.471200	13
**12**	2.50E-06	6.23E-06	< 1e-07	20.6	107.88	0.19	NRXN3	neurexin 3	9369	NM_004796	Hs.368307	18
**13**	2.70E-06	6.23E-06	2.00E-04	27.35	178.47	0.15	WSCD2	WSC domain containing 2	9671	BE962770	Hs.143591	12
**14**	4.61E-05	9.88E-05	2.00E-04	29.38	537.93	0.055	SPRR3	small proline rich protein 3	6707	BF575466	Hs.139322	13

**15**	6.99E-05	0.00014	< 1e-07	11.52	50.78	0.23	SUGCT	succinyl-CoA:glutarate-CoA transferase	79783	NM_024728	Hs.586313	15
**16**	8.72E-05	0.000164	< 1e-07	123.37	265.04	0.47	PSME3	proteasome activator subunit 3	10197	AA758755	Hs.152978	12
**17**	0.000201	0.000338	8.00E-04	10	386.13	0.026	SPRR1A	small proline rich protein 1A	6698	AI923984	Hs.46320	15
**18**	0.0002028	0.000338	0.0012	36.63	106.86	0.34	PART1	prostate androgen-regulated transcript 1 (non-protein coding)	25859	AI770098	Hs.146312	13
**19**	0.000342	0.00054	9.00E-04	20.38	459.07	0.044	SPRR1B	small proline rich protein 1B	6699	NM_003125	Hs.1076	17
**20**	0.000362	0.000543	0.001	19.06	98.14	0.19	THBS1	thrombospondin 1	7057	AW956580	Hs.164226	11
**21**	0.0005583	0.000798	0.0012	28.42	87.52	0.32	FAM124A	family with sequence similarity 124 member A	220108	AA151659	Hs.71913	14
**22**	0.0012344	0.00168	0.0026	71.23	130.43	0.55	REXO2	RNA exonuclease 2	25996	BC003502	Hs.677190	13
**23**	0.0018535	0.00242	0.0043	60.61	170.95	0.35	SLC7A5	solute carrier family 7 member 5	8140	AB018009	Hs.513797	11
**24**	0.0019556	0.00244	0.0047	24.09	74.98	0.32	HRCT1	histidine rich carboxyl terminus 1	646962	AI521254	Hs.208081	13
**25**	0.0067395	0.00809	0.01	133.95	202.44	0.66	CREB3	cAMP responsive element binding protein 3	10488	AF029674	Hs.522110	18
**26**	0.0089333	0.0103	0.0093	28.14	43.41	0.65	TOR3A	torsin family 3 member A	64222	AJ299441	Hs.584957	20
**27**	0.0132142	0.0147	0.0176	48.59	89.81	0.54	ITGB3	integrin subunit beta 3	3690	M35999	Hs.218040	19
**28**	0.0291398	0.0312	0.0407	64.56	112.14	0.58	SSSCA1	Sjogren syndrome/scleroderma autoantigen 1	10534	NM_006396	Hs.25723	11

Depicted are the geometric measures of intensity (GMI) for the groups: TEF; tracheoesophageal fistula, E; esophagus, T; trachea, L; Lung. Foldchange and statistics represent the comparison of combined controls and tracheoesophageal fistula. Genes are ranked on their p-value of the univariate test. FDR corrected p-values are depicted in the adjacent column. Type of univariate test used: Two-sample T-test. Permutation p-values for significant genes were computed based on 10000 random permutations. Nominal significance level of each univariate test: 0.05. Confidence level of false discovery rate assessment: 80 %, Maximum allowed proportion of false-positive genes: 0.01.

S2b Mostly absent in TEF, present in Esophagus and Trachea

Rank	Parametric p-value	FDR	Permutation p-value	Geom mean of intensities in Controls (E + T)	Geom mean of intensities in TEF	Fold-change	Symbol	Name	EntrezID	Accession	UGCluster	Times absent in TEF
**1**	< 1e-07	3.80E-06	< 1e-07	849.62	199.05	4.27	PCOLCE	procollagen C-endopeptidase enhancer	5118	NM_002593	Hs.202097	13
**2**	2.00E-07	3.80E-06	< 1e-07	137.16	28.71	4.78	CCDC102B	coiled-coil domain containing 102B	79839	NM_024781	Hs.280781	15
**3**	2.00E-07	3.80E-06	< 1e-07	196.57	64.04	3.07	MYLIP	myosin regulatory light chain interacting protein	29116	NM_013262	Hs.484738	11
**4**	4.00E-07	5.70E-06	< 1e-07	62.98	22.11	2.85	EPOR	erythropoietin receptor	2057	M60459	Hs.631624	12
**5**	1.10E-06	1.25E-05	< 1e-07	442.41	113.83	3.89	HIST1H2BD	histone cluster 1 H2B family member d	3017	AL353759	Hs.591797	12
**6**	3.20E-06	3.04E-05	< 1e-07	40.2	15.38	2.61	BBS9	Bardet-Biedl syndrome 9	27241	U85995	Hs.372360	12
**7**	8.50E-06	6.20E-05	< 1e-07	47.69	17.8	2.68	CECR2	CECR2, histone acetyl-lysine reader	27443	BE551781	Hs.231895	12
**8**	8.70E-06	6.20E-05	< 1e-07	68.71	32.33	2.12	EPG5	ectopic P-granules autophagy protein 5 homolog	57724	AL833448	Hs.514843	15
**9**	1.12E-05	7.09E-05	< 1e-07	110.69	42.34	2.61	PTPRD	protein tyrosine phosphatase, receptor type D	5789	NM_002839	Hs.446083	11
**10**	2.22E-05	0.000127	3.00E-04	101.66	25.41	4	HSPD1	heat shock protein family D (Hsp60) member 1	3329	BF965447	Hs.595053	15
**11**	2.77E-05	0.000144	< 1e-07	147	79.1	1.86	MAPKAPK5-AS1	MAPKAPK5 antisense RNA 1	51275	NM_016534	Hs.333120	17
**12**	3.03E-05	0.000144	1.00E-04	428.09	181.65	2.36	MYL6B	myosin light chain 6B	140465	NM_002475	Hs.632731	11
**13**	5.26E-05	0.000203	2.00E-04	67.24	27.6	2.44	RNF125	ring finger protein 125	54941	NM_017831	Hs.633703	13
**14**	5.33E-05	0.000203	1.00E-04	42.83	17.33	2.47	CDC14A	cell division cycle 14A	8556	AF064103	Hs.127411	15

**15**	5.33E-05	0.000203	< 1e-07	133.15	59.12	2.25	LSAMP	limbic system associated membrane protein	4045	AI858493	Hs.26409	16
**16**	0.0001187	0.000414	1.00E-04	174.18	99.4	1.75	PLCXD1	phosphatidylinositol specific phospholipase C X domain containing 1	55344	NM_018390	Hs.522568	12
**17**	0.0001257	0.000414	3.00E-04	166.8	43.64	3.82	TRO	trophinin	7216	AF349719	Hs.633653	11
**18**	0.0001306	0.000414	4.00E-04	62.44	34.42	1.81	RAPGEF2	Rap guanine nucleotide exchange factor 2	9693	AL117397	Hs.744884	16
**19**	0.0001405	0.000422	5.00E-04	105.05	44.4	2.37	SNHG18	small nucleolar RNA host gene 18	1.01E+08	AI631964	Hs.34447	13
**20**	0.0002094	0.000597	4.00E-04	77.98	31.11	2.51	NFIB	nuclear factor I B	4781	U70862	Hs.644095	11
**21**	0.0002765	0.000751	8.00E-04	175.32	76.77	2.28	ELMO1	engulfment and cell motility 1	9844	NM_014800	Hs.434989	11
**22**	0.0004132	0.00107	4.00E-04	90.4	46.07	1.96	SLC24A1	solute carrier family 24-member 1	9187	NM_004727	Hs.173092	12
**23**	0.0005595	0.00139	5.00E-04	74.53	41.88	1.78	NR1H3	nuclear receptor subfamily 1 group H member 3	10062	NM_005693	Hs.438863	13
**24**	0.0006231	0.00148	0.0012	29.82	14.52	2.05	ZNF826P	zinc finger protein 826, pseudogene	664701	BC016785	Hs.631635	11
**25**	0.0018794	0.00428	0.0027	68.35	43.33	1.58	SPATA6	spermatogenesis associated 6	54558	NM_019073	Hs.538103	13
**26**	0.0024722	0.00542	0.0038	38.03	16.78	2.27	SRSF11	serine and arginine rich splicing factor 11	9295	T90915	Hs.479693	11
**27**	0.0028278	0.00588	0.0042	119.75	67.93	1.76	GIMAP2	GTPase, IMAP family member 2	26157	AI431931	Hs.647071	15
**28**	0.0028876	0.00588	0.0061	47.14	26.55	1.78	RBPJ	recombination signal binding protein for immunoglobulin kappa J region	3516	R45471	Hs.479396	12
**29**	0.0030308	0.00596	0.0047	49.96	28.19	1.77	H2BFXP	H2B histone family member X, pseudogene	767811	H09657	Hs.496530	14
**30**	0.003287	0.00625	0.0056	65.2	27.61	2.36	CCDC88C	coiled-coil domain containing 88C	440193	AB040942	Hs.525536	13

**31**	0.0041398	0.00761	0.0044	30.38	19.13	1.59	LRRC27	leucine rich repeat containing 27	80313	AK098652	Hs.119897	13
**32**	0.0053434	0.00952	0.0081	50.96	29.83	1.71	HAUS5	HAUS augmin like complex subunit 5	23354	AA845355	Hs.7426	13
**33**	0.0067456	0.0115	0.0111	51.57	26.73	1.93	LOC202181	SUMO interacting motifs containing 1 pseudogene	202181	NM_024651	Hs.189914	11
**34**	0.0068891	0.0115	0.0129	37.18	15.06	2.47	CASC1	cancer susceptibility 1	55259	NM_018272	Hs.407771	11
**35**	0.0080378	0.0131	0.01	74.24	44.81	1.66	KHDRBS1	KH RNA binding domain containing, signal transduction associated 1	10657	AW592227	Hs.445893	11
**36**	0.0087371	0.0138	0.0128	62.16	31.93	1.95	GLCCI1	glucocorticoid induced 1	113263	AC006042	Hs.131673	13
**37**	0.0133789	0.0206	0.0166	34.63	19.31	1.79	TNPO1	transportin 1	3842	AA639220	Hs.482497	11
**38**	0.0246895	0.0352	0.0347	342.41	189.86	1.8	DEPP1	DEPP1, autophagy regulator	11067	AL136653	Hs.93675	15
**39**	0.0337943	0.0455	0.0464	78.14	30.66	2.55	C9orf135	chromosome 9 open reading frame 135	138255	AI768674	Hs.444459	12
**40**	0.0343559	0.0455	0.0438	140.3	84.51	1.66	NIPBL	NIPBL, cohesin loading factor	25836	AW272262	Hs.481927	11

Depicted are the geometric measures of intensity (GMI) for the groups: TEF; tracheoesophageal fistula, E; esophagus, T; trachea, L; Lung. Foldchange and statistics represent the comparison of combined controls and tracheoesophageal fistula. Genes are ranked on their p-value of the univariate test. FDR corrected p-values are depicted in the adjacent column. Type of univariate test used: Two-sample T-test. Permutation p-values for significant genes were computed based on 10000 random permutations. Nominal significance level of each univariate test: 0.05. Confidence level of false discovery rate assessment: 80 %, Maximum allowed proportion of false-positive genes: 0.01.
