# Supplementary material for: Histological, immunohistochemical and transcriptomic characterization of human tracheoesophageal fistulas
Source: PLoS One. 2020 Nov 17;15(11):e0242167. doi: 10.1371/journal.pone.0242167 (PMC7671559; doi:10.1371/journal.pone.0242167)
Supplement: S3 File — (PDF) [file pone.0242167.s003.pdf]

S3 File: DEGs in WNT signaling, TGFB-signaling, Hedgehog signaling and Retinol metabolism

Parametric P-Value	FDR	Permutation p-value	GMI in Esophagus	GMI in TEF	GMI in Lung	GMI in Trachea	Symbol	Name	EntrezID	Pathway	Pairwise significant
< 1e-07	< 1e-07	< 1e-07	154.3	83.47	534.99	206.26	*FZD2*	frizzled class receptor 2	2535	Wnt signaling pathway	(2, 1), (1, 3), (2, 3), (2, 4), (4, 3)
5.00E-07	1.66E-05	< 1e-07	85.44	174.67	76.38	69.67	*CAMK2G*	calcium/calmodulin dependent protein kinase II gamma	818	Wnt signaling pathway	(1, 2), (3, 2), (4, 2)
5.00E-07	1.66E-05	< 1e-07	185.79	96.67	305.15	215.59	*PRICKLE1*	prickle planar cell polarity protein 1	144165	Wnt signaling pathway	(2, 1), (2, 3), (2, 4)
7.00E-07	1.66E-05	2.00E-04	242.34	192.22	798.57	317.43	*PTCH1*	patched 1	5727	Sonic Hedgehog (Shh) Pathway	(1, 3), (2, 3), (2, 4), (4, 3)
1.40E-06	2.77E-05	< 1e-07	158.48	605.48	114	256.87	*DAAM2*	dishevelled associated activator of morphogenesis 2	23500	Wnt signaling pathway	(1, 2), (3, 2), (4, 2)
1.50E-06	2.77E-05	1.00E-04	135.86	52.78	94.31	365.88	*DKK2*	dickkopf WNT signaling pathway inhibitor 2	27123	Wnt signaling pathway	(2, 1), (1, 4), (2, 3), (2, 4), (3, 4)
2.10E-06	3.49E-05	1.00E-04	654.43	131.45	3664.29	267.71	*WIF1*	WNT inhibitory factor 1	11197	Wnt signaling pathway	(2, 1), (1, 3), (2, 3), (4, 3)
2.70E-06	4.07E-05	2.00E-04	239.01	109.71	317.76	175.7	*SMAD7*	SMAD family member 7	4092	TGF beta signaling pathway	(2, 1), (2, 3), (2, 4), (4, 3)
5.80E-06	7.53E-05	< 1e-07	211.85	226.86	523.25	116	*AXIN2*	axin 2	8313	Wnt signaling pathway	(1, 3), (4, 1), (2, 3), (4, 2), (4, 3)
5.90E-06	7.53E-05	< 1e-07	49.71	30.45	101.55	64.78	*FZD1*	frizzled class receptor 1	8321	Wnt signaling pathway	(2, 1), (1, 3), (2, 3), (2, 4)
8.20E-06	9.72E-05	1.00E-04	122.16	51.11	253.99	108.75	*ZFYVE16*	zinc finger FYVE-type containing 16	9765	TGF-beta signaling pathway	(2, 1), (2, 3), (2, 4), (4, 3)
1.05E-05	0.000114	6.00E-04	309.57	93.86	295.75	143.01	*SMAD6*	SMAD family member 6	4091	TGF-beta signaling pathway	(2, 1), (4, 1), (2, 3), (4, 3)

1.10E-05	0.000114	< 1e-07	228.6	594.8	116.87	347.24	*MYC*	MYC proto-oncogene, bHLH transcription factor	4609	TGF-beta signaling pathway	(1, 2), (3, 2), (3, 4)
1.27E-05	0.000117	1.00E-04	382.71	234.75	278.55	791.94	*TCF7*	transcription factor 7	6932	Wnt signaling pathway	(2, 1), (1, 4), (2, 4), (3, 4)
1.39E-05	0.000121	< 1e-07	290.04	879.63	3579.82	410.03	*ADH1B*	alcohol dehydrogenase 1B (class I), beta polypeptide	125	Retinol metabolism	(1, 2), (1, 3), (2, 3), (4, 3)
4.49E-05	0.000355	5.00E-04	132.08	115.47	39.87	578.5	*SFRP1*	secreted frizzled related protein 1	6422	Wnt signaling pathway	(3, 1), (1, 4), (3, 2), (2, 4), (3, 4)
5.61E-05	0.00036	< 1e-07	206.61	106.32	241.13	316.37	*LEF1*	lymphoid enhancer binding factor 1	51176	Wnt signaling pathway	(2, 1), (2, 3), (2, 4)
6.73E-05	0.000414	7.00E-04	136.42	57.08	202.35	104.69	*BMP2*	bone morphogenetic protein 2	650	TGF-beta signaling pathway	(2, 1), (2, 3)
9.44E-05	0.000544	2.00E-04	87.57	46.26	178.01	66.14	*TGFB2*	transforming growth factor beta 2	7042	TGF-beta signaling pathway	(2, 1), (2, 3), (4, 3)
9.51E-05	0.000544	0.0012	961.6	316.42	282.03	1234.35	*THBS2*	thrombospondin 2	7058	TGF-beta signaling pathway	(2, 1), (3, 1), (2, 4), (3, 4)
9.90E-05	0.000548	1.00E-04	258.91	252.54	505.12	168.8	*PPP2R5A*	protein phosphatase 2 regulatory subunit B'alpha	5525	Wnt signaling pathway	(1, 3), (2, 3), (4, 2), (4, 3)
0.0001039	0.000556	3.00E-04	80.82	67.38	167.04	107.36	*VANGL2*	VANGL planar cell polarity protein 2	57216	Wnt signaling pathway	(1, 3), (2, 3), (2, 4)
0.0001603	0.000806	3.00E-04	127.03	93.85	215.4	133.83	*SIAH1*	siah E3 ubiquitin protein ligase 1	6477	Wnt signaling pathway	(1, 3), (2, 3), (4, 3)
0.000168	0.00082	1.00E-04	50.3	85.99	34.6	53.62	*WNT9A*	Wnt family member 9A	7483	Wnt signaling pathway	(1, 2), (3, 2), (4, 2)
0.00066	0.00267	0.0036	132.95	175.41	83.19	117.93	*RETSAT*	retinol saturase	54884	Retinol metabolism	(3, 2), (4, 2)
0.0007792	0.00308	0.0015	79.44	64.48	176.17	64.48	*SMAD4*	SMAD family member 4	4089	TGF-beta signaling pathway,	(1, 3), (2, 3), (4, 3)

										Wnt signaling pathway	
0.0008584	0.00331	9.00E-04	203.73	154.05	392.09	349.05	*SMAD5*	SMAD family member 5	4090	TGF-beta signaling pathway	(2, 3), (2, 4)
0.0012004	0.00446	0.0039	58.95	148.94	37.03	334.72	*FST*	follistatin	10468	TGF-beta signaling pathway	(1, 4), (3, 2), (3, 4)
0.0023377	0.00808	0.0035	125.93	206.39	113.76	157.89	*THBS3*	thrombospondin 3	7059	TGF-beta signaling pathway	(1, 2), (3, 2)
0.0035129	0.0117	0.0063	188.17	134.05	294.03	228.97	*NFAT5*	nuclear factor of activated T cells 5	10725	Wnt signaling pathway	(2, 3), (2, 4)
0.0036445	0.0119	0.005	765.78	709.33	513.69	1594.27	*GAS1*	growth arrest specific 1	2619	Hedgehog signaling pathway	(1, 4), (2, 4), (3, 4)
0.0051197	0.016	0.0111	185.38	366.24	209.55	193.97	*PRKCA*	protein kinase C alpha	5578	Wnt signaling pathway	(1, 2), (3, 2), (4, 2)
0.0053255	0.0164	0.0057	65.62	60.68	121.86	74.7	*SMAD1*	SMAD family member 1	4086	TGF-beta signaling pathway	(1, 3), (2, 3)
0.0056393	0.017	0.0086	97.89	101.51	36.8	77.14	*FZD10*	frizzled class receptor 10	11211	Wnt signaling pathway	(3, 1), (3, 2)
0.0059177	0.0175	0.0076	112.62	116.29	82.32	198.42	*NFATC1*	nuclear factor of activated T cells 1	4772	Wnt signaling pathway	(1, 4), (3, 2), (2, 4), (3, 4)
0.0066806	0.0191	0.0111	230.64	160.55	374.22	228.88	*EP300*	E1A binding protein p300	2033	TGF beta signaling pathway	(2, 3)
0.0113034	0.0313	0.0177	675.1	392.04	1447.07	1108.1	*ID3*	inhibitor of DNA binding 3, HLH protein	3399	TGF-beta signaling pathway	(2, 3), (2, 4)
0.0164518	0.0433	0.0191	161.15	317.84	271.97	208.51	*ROCK1*	Rho associated coiled-coil containing protein kinase 1	6093	Wnt signaling pathway	(1, 2)
0.0203857	0.0521	0.0159	202.12	307.33	251.35	198.45	*BMPR1A*	bone morphogenetic protein receptor type 1A	657	TGF-beta signaling pathway	(1, 2), (4, 2)

0.0217747	0.0548	0.0276	193.37	156.13	333.89	140.78	*CSNK1A1*	casein kinase 1 alpha 1	1452	Wnt signaling pathway	(2, 3), (4, 3)
0.0239799	0.0594	0.03	1068.42	915.68	1568.27	1127.89	*CTBP2*	C-terminal binding protein 2	1488	Wnt signaling pathway	(2, 3)
0.0288689	0.0705	0.0278	42.09	53.99	35.14	43.63	*FRAT1*	FRAT1, WNT signaling pathway regulator	10023	Wnt signaling pathway	(3, 2)
0.0294226	0.0708	0.0358	105.14	187.01	94.92	165.21	LTBP1	latent transforming growth factor beta binding protein 1	4052	TGF-beta signaling pathway	(3, 2)
0.030409	0.0715	0.0351	271.83	215.22	289.53	356.64	CCND3	cyclin D3	896	Wnt signaling pathway	(2, 4)
0.033589	0.0765	0.0481	345.83	323.49	720.25	477.51	TBL1XR1	transducin beta like 1 X-linked receptor 1	79718	Wnt signaling pathway	(2, 3)
0.0339696	0.0765	0.0477	178.87	131.12	205.62	293.34	CAMK2D	calcium/calmodulin dependent protein kinase II delta	817	Wnt signaling pathway	(2, 4)
0.0341245	0.0765	0.0438	86.15	91.46	43.61	82.77	PLCB4	phospholipase C beta 4	5332	Wnt signaling pathway	(3, 2)
0.0394099	0.0872	0.0584	224.38	400.48	244.9	311.99	FZD7	frizzled class receptor 7	8324	Wnt signaling pathway	(1, 2), (3, 2)
0.0408846	0.0893	0.0392	182.22	209.86	131.78	165.95	PPP2R5C	protein phosphatase 2 regulatory subunit B'gamma	5527	Wnt signaling pathway	(3, 2)
0.0477834	0.1	0.0683	883.94	1268.52	735.7	1230.58	RHOA	ras homolog family member A	387	Wnt signaling pathway	(3, 2)

Depicted are the geometric measures of intensity (GMI) for the groups: (1) Esophagus, (2) TEF, (3) Lung and (4) Trachea. Pairwise significance is depicted in the last column. The GMI intensity boxes are labeled in a color scale from red (low) to green (high). For example: Highly upregulated in TEF is the expression of DAAM2 compared to all control tissue types and downregulated is the expression of *BMP2*. Genes are ranked on their pairwise class comparison according to the random variance t-test analysis. The columns are sorted by the parametric P-value, the false discovery rate (FDR) and the univariate permutation p-value. Genes and pathways as described in the Kyoto encyclopedia of genes and genomes; https://www.genome.jp/kegg/kegg2.html or REACTOME at https://reactome.org/), namely the TGF-beta / BMP signaling pathway (N00063), Wnt-signaling pathway (N00056), Hedgehog signaling pathway (N00062) and Retinol metabolism (ko00830).
