# Supplementary material for: Histological, immunohistochemical and transcriptomic characterization of human tracheoesophageal fistulas
Source: PLoS One. 2020 Nov 17;15(11):e0242167. doi: 10.1371/journal.pone.0242167 (PMC7671559; doi:10.1371/journal.pone.0242167)
Supplement: S4 File — (PDF) [file pone.0242167.s004.pdf]

S4 File: Smooth muscle contraction gene expression

Parametric P-Value	FDR	Permutation p-value	GMI in Esophagus	GMI in TEF	GMI in Lung	GMI in Trachea	Symbol	Name	EntrezID	Pairwise significant
< 1e-07	< 1e-07	< 1e-07	101.81	1719.36	80.43	141.73	KCNMB1	potassium calcium-activated channel subfamily M regulatory beta subunit 1	3779	(1, 2), (3, 2), (4, 2)
< 1e-07	< 1e-07	< 1e-07	702.63	5626.32	550.08	568.76	MYL9	myosin light chain 9	10398	(1, 2), (3, 2), (4, 2)
< 1e-07	< 1e-07	< 1e-07	16.95	207.2	31.52	14.03	MYLK	myosin light chain kinase	4638	(1, 2), (3, 2), (4, 2), (4, 3)
< 1e-07	< 1e-07	< 1e-07	17.19	114.04	10	25.83	KCNMA1	potassium calcium-activated channel subfamily M alpha 1	3778	(1, 2), (3, 2), (4, 2), (3, 4)
< 1e-07	< 1e-07	1.00E-04	975.48	12547.21	604.38	1270.12	ACTG2	actin, gamma 2, smooth muscle, enteric	72	(1, 2), (3, 2), (4, 2)
< 1e-07	< 1e-07	< 1e-07	163.56	1452.6	185.19	248.5	PPP1R12B	protein phosphatase 1 regulatory subunit 12B	4660	(1, 2), (3, 2), (4, 2)
< 1e-07	< 1e-07	< 1e-07	11.81	124.28	17.39	11.54	PLA2G5	phospholipase A2 group V	5322	(1, 2), (3, 2), (4, 2)
< 1e-07	< 1e-07	< 1e-07	530.66	136.15	901.36	707.05	EDNRA	endothelin receptor type A	1909	(2, 1), (2, 3), (2, 4)
< 1e-07	< 1e-07	< 1e-07	178.36	108.99	739.38	329.51	GUCY1A2	guanylate cyclase 1 soluble subunit alpha 2	2977	(1, 3), (2, 3), (2, 4), (4, 3)
< 1e-07	< 1e-07	< 1e-07	98.16	688.16	134	82.2	PPP1R14A	protein phosphatase 1 regulatory inhibitor subunit 14A	94274	(1, 2), (3, 2), (4, 2)
< 1e-07	< 1e-07	< 1e-07	98.68	384.68	47.51	68.7	RAMP1	receptor activity modifying protein 1	10267	(1, 2), (3, 2), (4, 2)
< 1e-07	< 1e-07	< 1e-07	71.93	32.64	112.03	52.43	CACNA1D	calcium voltage-gated channel subunit alpha1 D	776	(2, 1), (1, 3), (2, 3), (2, 4),
< 1e-07	< 1e-07	< 1e-07	23.15	364.97	13.26	11.06	MYH11	myosin heavy chain 11	4629	(1, 2), (3, 2), (4, 2)
< 1e-07	4.64E-07	< 1e-07	106.42	56.13	222.38	120.34	BRAF	B-Raf proto-oncogene, serine/threonine kinase	673	(2, 1), (1, 3), (2, 3), (2, 4),
2.00E-07	8.67E-07	< 1e-07	139.39	77.34	340.61	297.23	AGTR1	angiotensin II receptor type 1	185	(2, 1), (1, 3), (1, 4), (2, 3),
5.00E-07	2.03E-06	< 1e-07	358.41	1156.18	520.13	373.31	PPP1R12A	protein phosphatase 1 regulatory subunit 12A	4659	(1, 2), (3, 2), (4, 2)
1.10E-06	4.21E-06	< 1e-07	95.83	448.06	84.23	90.9	MRVI1	murine retrovirus integration site 1 homolog	10335	(1, 2), (3, 2), (4, 2)
4.60E-06	1.66E-05	< 1e-07	19.2	16.55	52.7	28.98	KCNMB2	potassium calcium-activated channel subfamily M regulatory beta subunit 2	10242	(1, 3), (2, 3), (2, 4), (4, 3)
1.96E-05	6.71E-05	< 1e-07	231.23	129.46	688.34	179.96	CALCRL	calcitonin receptor like receptor	10203	(1, 3), (2, 3), (4, 3)

2.55E-05	8.29E-05	< 1e-07	22.97	59.91	24.54	23.74	CACNA1C	calcium voltage-gated channel subunit alpha1 C	775	(1, 2), (3, 2), (4, 2)
4.97E-05	0.000154	4.00E-04	45.4	43.04	215.4	40.03	PRKCB	protein kinase C beta	5579	(1, 3), (2, 3), (4, 3)
6.02E-05	0.000178	< 1e-07	74.61	335.71	40.69	33.89	PLA2G2A	phospholipase A2 group IIA	5320	(1, 2), (3, 2), (4, 2)
6.66E-05	0.000188	2.00E-04	158.97	767.9	176.83	277.88	CALD1	caldesmon 1	800	(1, 2), (3, 2), (4, 2)
0.0001223	0.000318	4.00E-04	188.83	136.81	287.12	197.74	GNA13	G protein subunit alpha 13	10672	(1, 3), (2, 3), (2, 4)
0.0001225	0.000318	6.00E-04	421.2	181.65	336.77	435.08	MYL6B	myosin light chain 6B	140465	(2, 1), (2, 3), (2, 4)
0.0003025	0.000756	6.00E-04	201.06	127.78	454.64	149.92	ITPR3	inositol 1,4,5-trisphosphate receptor type 3	3710	(1, 3), (2, 3), (4, 3)
0.0005834	0.0014	6.00E-04	121.28	80.92	165.68	84.35	RAF1	Raf-1 proto-oncogene, serine/threonine kinase	5894	(2, 1), (2, 3), (4, 3)
0.0006163	0.00143	0.003	149.43	103.47	27.62	427.29	AVPR1A	arginine vasopressin receptor 1A	552	(3, 1), (3, 2), (2, 4), (3, 4)
0.0007979	0.00179	0.0018	101.2	358.39	156.03	190.22	ACTA2	actin, alpha 2, smooth muscle, aorta	59	(1, 2), (3, 2)
0.0011375	0.00246	9.00E-04	312.3	178.87	358.94	407.35	GNAS	GNAS complex locus	2778	(2, 1), (2, 3), (2, 4)
0.003781	0.00793	0.0096	224.28	376.38	77.12	164.01	CALM1	calmodulin 1	801	(3, 2)
0.0051197	0.0104	0.0111	185.38	366.24	209.55	193.97	PRKCA	protein kinase C alpha	5578	(1, 2), (3, 2), (4, 2)
0.0089887	0.0177	0.0136	83.3	144.63	75.31	68.54	ITPR1	inositol 1,4,5-trisphosphate receptor type 1	3708	(3, 2), (4, 2)
0.0164518	0.0315	0.0191	161.15	317.84	271.97	208.51	ROCK1	Rho associated coiled-coil containing protein kinase 1	6093	(1, 2)
0.0341245	0.0634	0.0438	86.15	91.46	43.61	82.77	PLCB4	phospholipase C beta 4	5332	(3, 2)
0.0477834	0.0863	0.0683	883.94	1268.52	735.7	1230.58	RHOA	ras homolog family member A	387	(3, 2)

Depicted are the geometric measures of intensity (GMI) for the groups: (1) Esophagus, (2) TEF, (3) Lung and (4) Trachea. Pairwise significance is depicted in the last column. The GMI intensity boxes are labeled in a color scale from red (low) to green (high). For example: Highly upregulated in TEF is the expression of *MYL9* compared to all control tissue types and downregulated is the expression of *CACNA1D*. Genes are ranked on their pairwise class comparison according to the random variance t-test analysis. The columns are sorted by the parametric P-value, the false discovery rate (FDR) and the univariate permutation p-value.
