# Supplementary material for: Histological, immunohistochemical and transcriptomic characterization of human tracheoesophageal fistulas
Source: PLoS One. 2020 Nov 17;15(11):e0242167. doi: 10.1371/journal.pone.0242167 (PMC7671559; doi:10.1371/journal.pone.0242167)
Supplement: S5 File — (PDF) [file pone.0242167.s005.pdf]

S5 File: Neuronal marker genes

Parametric P-Value	FDR	Permutation p-value	GMI in Esophagus	GMI in TEF	GMI in Lung	GMI in Trachea	Symbol	Name	EntrezID	Pairwise significant
< 1e-07	< 1e-07	< 1e-07	101.81	1719.36	80.43	141.73	KCNMB1	potassium calcium-activated channel subfamily M regulatory beta subunit 1	3779	(1, 2), (3, 2), (4, 2)
< 1e-07	< 1e-07	< 1e-07	41.93	223.97	18.37	19.61	KCND3	potassium voltage-gated channel subfamily D member 3	3752	(1, 2), (3, 1), (3, 2), (4, 2)
< 1e-07	< 1e-07	< 1e-07	17.19	114.04	10	25.83	KCNMA1	potassium calcium-activated channel subfamily M alpha 1	3778	(1, 2), (3, 2), (4, 2), (3, 4)
< 1e-07	< 1e-07	< 1e-07	121.71	1177.89	31.89	60.11	PCP4	Purkinje cell protein 4	5121	(1, 2), (3, 1), (3, 2), (4, 2)
< 1e-07	< 1e-07	< 1e-07	71.93	32.64	112.03	52.43	CACNA1D	calcium voltage-gated channel subunit alpha1 D	776	(2, 1), (1, 3), (2, 3), (2, 4), (4, 3)
< 1e-07	< 1e-07	3.00E-04	306.01	118.88	1288.1	125.17	NDNF	neuron derived neurotrophic factor	79625	(2, 1), (1, 3), (4, 1), (2, 3), (4, 3)
< 1e-07	< 1e-07	< 1e-07	46.13	121.24	22.31	57.61	KCNB1	potassium voltage-gated channel subfamily B member 1	3745	(1, 2), (3, 1), (3, 2), (4, 2), (3, 4)
< 1e-07	< 1e-07	< 1e-07	34.79	119.19	29.33	25.6	KCNQ4	potassium voltage-gated channel subfamily Q member 4	9132	(1, 2), (3, 2), (4, 2)
< 1e-07	8.70E-07	< 1e-07	47.32	225.45	32.3	50.7	CHRM3	cholinergic receptor muscarinic 3	1131	(1, 2), (3, 2), (4, 2)
< 1e-07	8.70E-07	< 1e-07	59.58	182.28	35.6	161.96	SULF2	sulfatase 2	55959	(1, 2), (1, 4), (3, 2), (3, 4)
2.00E-07	1.58E-06	< 1e-07	50.09	231.73	59.49	75.32	KCNH2	potassium voltage-gated channel subfamily H member 2	3757	(1, 2), (3, 2), (4, 2)
1.70E-06	1.23E-05	< 1e-07	38.61	214.18	21.2	44.42	VIP	vasoactive intestinal peptide	7432	(1, 2), (3, 2), (4, 2)
2.50E-06	1.67E-05	2.00E-04	66.72	52.48	150.35	84.66	KCNK17	potassium two pore domain channel subfamily K member 17	89822	(1, 3), (2, 3), (2, 4), (4, 3)
4.60E-06	2.86E-05	< 1e-07	19.2	16.55	52.7	28.98	KCNMB2	potassium calcium-activated channel subfamily M regulatory beta subunit 2	10242	(1, 3), (2, 3), (2, 4), (4, 3)
5.70E-06	3.31E-05	1.00E-04	30.6	84.02	27.01	37.54	GDNF	glial cell derived neurotrophic factor	2668	(1, 2), (3, 2), (4, 2)
1.06E-05	5.76E-05	3.00E-04	59.42	111.88	24.61	354.71	SOX10	SRY-box 10	6663	(1, 4), (3, 2), (2, 4), (3, 4)
1.16E-05	5.94E-05	2.00E-04	139.19	36.83	477.87	18.09	KCNJ15	potassium voltage-gated channel subfamily J member 15	3772	(2, 1), (4, 1), (2, 3), (4, 3)
2.55E-05	0.000123	< 1e-07	22.97	59.91	24.54	23.74	CACNA1C	calcium voltage-gated channel subunit alpha1 C	775	(1, 2), (3, 2), (4, 2)

3.29E-05	0.000151	< 1e-07	12.85	30.4	17.27	16.82	CHRNA5	cholinergic receptor nicotinic alpha 5 subunit	1138	(1, 2), (3, 2), (4, 2)
4.25E-05	0.000185	5.00E-04	36.4	110.77	64.06	13.12	CACNB2	calcium voltage-gated channel auxiliary subunit beta 2	783	(1, 2), (4, 2), (4, 3)
6.28E-05	0.00026	1.00E-04	37.36	21.96	58.43	41.56	CACNA1G	calcium voltage-gated channel subunit alpha1 G	8913	(2, 1), (2, 3), (2, 4)
6.68E-05	0.000264	< 1e-07	1995.9	1531.37	2409.01	2658.72	GABARAP	GABA type A receptor-associated protein	11337	(2, 1), (2, 3), (2, 4)
0.0001162	0.00044	9.00E-04	25.07	53.87	17.43	118.47	GRIK3	glutamate ionotropic receptor kainate type subunit 3	2899	(1, 2), (1, 4), (3, 2), (2, 4), (3, 4)
0.0001682	0.00061	0.001	197.8	185.32	137.69	783.91	RELN	reelin	5649	(1, 4), (2, 4), (3, 4)
0.0002056	0.000715	3.00E-04	14.68	44.9	17.69	18.93	KCNJ3	potassium voltage-gated channel subfamily J member 3	3760	(1, 2), (3, 2), (4, 2)
0.0003214	0.00108	0.0017	47.23	25.06	12.49	109.63	NPY1R	neuropeptide Y receptor Y1	4886	(3, 1), (2, 4), (3, 4)
0.0003645	0.00117	0.0016	245.68	114.11	32.62	213.24	KCNAB1	potassium voltage-gated channel subfamily A member regulatory beta subunit 1	7881	(3, 1), (3, 2), (3, 4)
0.0011664	0.00362	0.0011	18.15	27.09	39.52	64.97	SCN9A	sodium voltage-gated channel alpha subunit 9	6335	(1, 3), (1, 4), (2, 4)
0.0014221	0.00427	0.0047	117.38	56.57	37.34	395.77	NTS	neurotensin	4922	(2, 4), (3, 4)
0.0016035	0.00465	0.0031	31.93	41.82	19.73	36.22	RASGRF2	Ras protein specific guanine nucleotide releasing factor 2	5924	(3, 2), (3, 4)
0.0024806	0.00696	0.0032	77.1	55.01	123.73	86.75	KCNK5	potassium two pore domain channel subfamily K member 5	8645	(2, 3)
0.0028401	0.00761	0.0069	55.97	171.56	36.38	168.73	NPY	neuropeptide Y	4852	(1, 2), (3, 2), (3, 4)
0.0028876	0.00761	0.0036	58.73	61.94	21.25	23.23	CARTPT	CART prepropeptide	9607	(3, 1), (3, 2), (4, 2)
0.0031309	0.00775	0.0036	36.46	32.68	17.68	34.53	GRM7	glutamate metabotropic receptor 7	2917	(3, 1), (3, 2), (3, 4)
0.0032085	0.00775	0.0056	103.23	96.35	195.57	68.85	KCNS3	potassium voltage-gated channel modifier subfamily S member 3	3790	(1, 3), (2, 3), (4, 3)
0.0032875	0.00775	0.0072	70.51	53.82	150.49	28.41	EDN3	endothelin 3	1908	(2, 3), (4, 3)
0.0032959	0.00775	0.0074	43.59	73.6	82.01	33.77	KCNK3	potassium two pore domain channel subfamily K member 3	3777	(1, 2), (1, 3), (4, 2), (4, 3)
0.00362	0.00829	0.0045	37.27	53.72	29.43	40.48	DRD5	dopamine receptor D5	1816	(3, 2)
0.003761	0.00839	0.0085	39.83	26.56	13.77	70.86	SCN3A	sodium voltage-gated channel alpha subunit 3	6328	(3, 1), (3, 2), (2, 4), (3, 4)
0.0045428	0.00988	0.0111	138.07	348.57	75.72	370.8	NR4A2	nuclear receptor subfamily 4 group A member 2	4929	(3, 2), (3, 4)

0.0050354	0.0107	0.0045	72.81	98.05	142.89	67.58	LHX6	LIM homeobox 6	26468	(1, 3), (2, 3), (4, 2), (4, 3)
0.0055724	0.0115	0.0078	315.51	355.56	154.17	274.38	CACNA2D1	calcium voltage-gated channel auxiliary subunit alpha2delta 1	781	(3, 1), (3, 2)
0.0058606	0.0119	0.0096	71.18	114	58.63	137.87	HTRA3	HtrA serine peptidase 3	94031	(1, 4), (3, 2), (3, 4)
0.0061441	0.0121	0.009	463.28	541.33	1421.79	582.9	EDNRB	endothelin receptor type B	1910	(1, 3), (2, 3), (4, 3)
0.007008	0.0135	0.012	20.58	93.27	18.45	21.85	HTR2B	5-hydroxytryptamine receptor 2B	3357	(1, 2), (3, 2), (4, 2)
0.0086441	0.0163	0.0104	266.51	288.79	141.34	339.53	KCNJ8	potassium voltage-gated channel subfamily J member 8	3764	(3, 1), (3, 2), (3, 4)
0.0088356	0.0164	0.0208	26.73	59.52	28.83	112.96	CHRNA3	cholinergic receptor nicotinic alpha 3 subunit	1136	(1, 2), (1, 4), (3, 2), (3, 4)
0.0101239	0.0181	0.0134	25.13	39.88	18.28	48.55	RET	ret proto-oncogene	5979	(3, 2), (3, 4)
0.0102044	0.0181	0.016	83.46	144.35	67.09	130.3	KCNE4	potassium voltage-gated channel subfamily E regulatory subunit 4	23704	(3, 2)
0.0131869	0.0229	0.0224	25.1	55.19	59.36	18.47	KCNN2	potassium calcium-activated channel subfamily N member 2	3781	(4, 2), (4, 3)
0.0139124	0.0237	0.0236	69.43	40.78	94.99	130.23	THSD7A	thrombospondin type 1 domain containing 7A	221981	(2, 3), (2, 4)
0.0148007	0.0248	0.0218	22.01	42.11	16.67	30.98	KCNN3	potassium calcium-activated channel subfamily N member 3	3782	(3, 2)
0.0199279	0.0327	0.0345	107.38	182.49	66.23	195.94	PENK	proenkephalin	5179	(3, 2), (3, 4)
0.0213773	0.0344	0.035	257.45	107.13	120.8	89.43	KCNE3	potassium voltage-gated channel subfamily E regulatory subunit 3	10008	(2, 1), (3, 1), (4, 1)
0.0249446	0.0395	0.0399	36.61	37.4	47.07	97.18	SCN2B	sodium voltage-gated channel beta subunit 2	6327	(1, 4), (2, 4)
0.0332277	0.0516	0.0447	158.45	360.41	308.76	188.3	FOXP2	forkhead box P2	93986	(1, 2)
0.0361313	0.0551	0.0304	85.7	105.43	116.59	171.35	GABBR1	gamma-aminobutyric acid type B receptor subunit 1	2550	(1, 4), (2, 4)
0.0386969	0.058	0.0903	23.32	14.87	15.87	33.6	KCND2	potassium voltage-gated channel subfamily D member 2	3751	(2, 4)
0.0425647	0.0628	0.0412	75.62	52.96	49.88	80.62	CACNA1A	calcium voltage-gated channel subunit alpha1 A	773	(2, 4), (3, 4)
0.0469809	0.0681	0.0802	14	19.7	11.62	33.43	TAC1	tachykinin precursor 1	6863	(3, 4)
0.0486302	0.0694	0.06	21.32	23.62	13.41	29.15	KCNIP1	potassium voltage-gated channel interacting protein 1	30820	(3, 2), (3, 4)

Depicted are the geometric measures of intensity (GMI) for the groups: (1) Esophagus, (2) TEF, (3) Lung and (4) Trachea. Pairwise significance is depicted in the last column. The GMI intensity boxes are labeled in a color scale from red (low) to green (high). For example: Highly upregulated in TEF is the expression of KCNMB*1* compared to all control tissue types. Genes are ranked on their pairwise class comparison according to the random variance t-test analysis. The columns are sorted by the parametric P-value, the false discovery rate (FDR) and the univariate permutation p-value.
