# Supplementary material for: Histological, immunohistochemical and transcriptomic characterization of human tracheoesophageal fistulas
Source: PLoS One. 2020 Nov 17;15(11):e0242167. doi: 10.1371/journal.pone.0242167 (PMC7671559; doi:10.1371/journal.pone.0242167)
Supplement: S6 File — (PDF) [file pone.0242167.s006.pdf]

S6 File: DEG ENS markers

Parametric p-value	FDR	Permutation p-value	Geom mean of intensities in Esophagus	Geom mean of intensities in TEF	Geom mean of intensities in Lung	Geom mean of intensities in Trachea	Symbol	Name	EntrezID	Pairwise significant
1.06E-05	9.12E-05	3.00E-04	59.42	111.88	24.61	354.71	SOX10	SRY-box 10	6663	(1, 4), (3, 2), (2, 4), (3, 4)
1.43E-05	9.12E-05	< 1e-07	45.27	112.35	22.06	236.7	L1CAM	L1 cell adhesion molecule	3897	(1, 2), (1, 4), (3, 2), (3, 4)
1.52E-05	9.12E-05	4.00E-04	64.13	60.16	179.51	68.47	HOXB5	homeobox B5	3215	(1, 3), (2, 3), (4, 3)
3.06E-05	0.000138	0.0016	444.01	110.69	495.09	240.58	TMEM100	transmembrane protein 100	55273	(2, 1), (2, 3), (2, 4)
4.93E-05	0.000177	8.00E-04	726.62	1518.57	514.52	977.97	TUBB3	tubulin beta 3 class III	10381	(1, 2), (3, 2), (3, 4)
0.0001172	0.000352	4.00E-04	221.82	336.31	158.6	2075.03	PLP1	proteolipid protein 1	5354	(1, 4), (2, 4), (3, 4)
0.0002121	0.000545	8.00E-04	32.87	36.88	13.82	84.43	FOXD3	forkhead box D3	27022	(3, 1), (1, 4), (3, 2), (2, 4), (3, 4)
0.000325	0.000731	0.0011	140.06	200.93	63.68	418.94	UCHL1	ubiquitin C-terminal hydrolase [illegible]	7345	(1, 4), (3, 2), (2, 4), (3, 4)
0.000543	0.00109	0.0017	74.99	127.51	52.33	613.57	PRPH	peripherin	5630	(1, 4), (3, 2), (2, 4), (3, 4)
0.000827	0.00149	0.0021	59.79	90.1	26.89	213	ELAVL4	ELAV like RNA binding protein 4	1996	(1, 4), (3, 2), (2, 4), (3, 4)
0.0061441	0.0101	0.009	463.28	541.33	1421.79	582.9	EDNRB	endothelin receptor type B	1910	(1, 3), (2, 3), (4, 3)
0.0101239	0.0152	0.0134	25.13	39.88	18.28	48.55	RET	ret proto-oncogene	5979	(3, 2), (3, 4)
0.0216935	0.03	0.0495	39.31	47.5	22.66	71.08	NEFH	neurofilament heavy	4744	(3, 2), (3, 4)

Depicted are the geometric measures of intensity (GMI) for the groups: (1) Esophagus, (2) TEF, (3) Lung and (4) Trachea. Pairwise significance is depicted in the last column. The GMI intensity boxes are labeled in a color scale from red (low) to green (high). For example: Highly upregulated in TEF is the expression of TUBB*3* compared to all control tissue types. Genes are ranked on their pairwise class comparison according to the random variance t-test analysis. The columns are sorted by the parametric P-value, the false discovery rate (FDR) and the univariate permutation p-value.
