# Supplementary material for: Histological, immunohistochemical and transcriptomic characterization of human tracheoesophageal fistulas
Source: PLoS One. 2020 Nov 17;15(11):e0242167. doi: 10.1371/journal.pone.0242167 (PMC7671559; doi:10.1371/journal.pone.0242167)
Supplement: S7 File — (PDF) [file pone.0242167.s007.pdf]

S7 File: DEG epithelial markers

Parametric p-value	FDR	Permutation p-value	Geom mean of intensities in Esophagus	Geom mean of intensities in TEF	Geom mean of intensities in Lung	Geom mean of intensities in Trachea	Symbol	Name	EntrezID	Pairwise significant
1.59E-05	6.59E-05	< 1e-07	63.91	129.35	61.5	92.86	NT5E	5'-nucleotidase ecto	4907	(1, 2), (3, 2)
3.02E-05	0.000117	2.00E-04	450.22	2046.19	28.19	86.86	KRT13	keratin 13	3860	(3, 1), (3, 2), (4, 2)
0.0002413	0.000636	3.00E-04	27.5	747.88	18.24	109.15	KRT14	keratin 14	3861	(1, 2), (3, 2)
0.000884	0.00197	0.0011	201.33	1090.4	50.54	65.21	KRT4	keratin 4	3851	(3, 2), (4, 2)
0.0039302	0.00712	0.0073	673.78	588.54	288.48	185.71	KRT8	keratin 8	3856	(4, 1), (3, 2), (4, 2)
0.0043801	0.00747	0.0096	263.56	1295.09	44.69	147.99	KRT5	keratin 5	3852	(3, 2)
0.0149558	0.0197	0.0266	28.51	91.52	35.46	83.81	ITGB4	integrin subunit beta 4	3691	(1, 2), (3, 2)

Depicted are the geometric measures of intensity (GMI) for the groups: (1) Esophagus, (2) TEF, (3) Lung and (4) Trachea. Pairwise significance is depicted in the last column. The GMI intensity boxes are labeled in a color scale from red (low) to green (high). For example: Highly upregulated in TEF is the expression of KRT*13* and KRT*5* compared to all control tissue types. Genes are ranked on their pairwise class comparison according to the random variance t-test analysis. The columns are sorted by the parametric P-value, the false discovery rate (FDR) and the univariate permutation p-value.
