# Supplementary material for: Histological, immunohistochemical and transcriptomic characterization of human tracheoesophageal fistulas
Source: PLoS One. 2020 Nov 17;15(11):e0242167. doi: 10.1371/journal.pone.0242167 (PMC7671559; doi:10.1371/journal.pone.0242167)
Supplement: S8 File — (PDF) [file pone.0242167.s008.pdf]

S8 File: DEG cartilage markers

Parametric p-value	FDR	Permutation p-value	Geom mean of intensities in Esophagus	Geom mean of intensities in TEF	Geom mean of intensities in Lung	Geom mean of intensities in Trachea	Symbol	Name	EntrezID	Pairwise significant
2.00E-06	1.54E-05	1.00E-04	96.55	80.19	230.89	178.61	SOX5	SRY-box 5	6660	(1, 3), (1, 4), (2, 3), (2, 4)
2.20E-06	1.54E-05	3.00E-04	68.94	57.92	48.69	188.99	FOXC2	forkhead box C2	2303	(1, 4), (2, 4), (3, 4)
1.03E-05	4.81E-05	0.0018	90.84	15.66	109.21	15.35	COL4A3	collagen type IV alpha 3 chain	1285	(2, 1), (4, 1), (2, 3), (4, 3)
8.44E-05	0.000295	3.00E-04	1395.93	1193.65	321.28	641.9	CTSB	cathepsin B	1508	(3, 1), (3, 2), (4, 2)
0.0007758	0.00217	0.0016	1930.34	1042.33	1262.95	3803.14	ITM2A	integral membrane protein 2A	9452	(2, 4), (3, 4)
0.0015151	0.00326	0.0012	142.37	176.73	103.78	102.48	FAM20B	FAM20B, glycosaminoglycan xylosylkinase	9917	(3, 2), (4, 2)
0.0016287	0.00326	0.002	189.23	256.33	113.93	153.72	CD151	CD151 molecule (Raph blood group)	977	(3, 2), (4, 2)
0.0059234	0.0104	0.0105	116.57	148.32	281.85	104.65	CD44	CD44 molecule (Indian blood group)	960	(1, 3), (2, 3), (4, 3)
0.008409	0.0131	0.0167	38.41	79.36	40.3	188.74	MIA	melanoma inhibitory activity	8190	(1, 4), (2, 4), (3, 4)
0.0117952	0.0165	0.023	42.1	95.07	36.94	208.95	FOXC1	forkhead box C1	2296	(1, 4), (3, 2), (3, 4)
0.0407292	0.0518	0.0716	21.01	18.72	34.78	71.38	MATN3	matrilin 3	4148	(2, 4)

Depicted are the geometric measures of intensity (GMI) for the groups: (1) Esophagus, (2) TEF, (3) Lung and (4) Trachea. Pairwise significance is depicted in the last column. The GMI intensity boxes are labeled in a color scale from red (low) to green (high). For example: Highly upregulated in TEF is the expression of ITGB*1* compared to all control tissue types. Genes are ranked on their pairwise class comparison according to the random variance t-test analysis. The columns are sorted by the parametric P-value, the false discovery rate (FDR) and the univariate permutation p-value. We used the cartilage markers ANXA6, CD44, CD151, ITM2A, FAM20B, FOXC1, FOXC2, SOX5 SOX6, SOX9, ACAN, CTSB, CHADL, CHAD, COL2A1, COL4A3, CRTAC1, DSPG3, IBSP, MATN1, MATN3, MATN4, MIA, OTOR, URB
