# Supplementary material for: Histological, immunohistochemical and transcriptomic characterization of human tracheoesophageal fistulas
Source: PLoS One. 2020 Nov 17;15(11):e0242167. doi: 10.1371/journal.pone.0242167 (PMC7671559; doi:10.1371/journal.pone.0242167)
Supplement: S9 File — (PDF) [file pone.0242167.s009.pdf]

S9 File: Details of antibodies used for immunohistochemistry

Antibody	Company	Dilution	Host	Antibody type	Incubation	Catalog number	Remarks
TTF1	Neomarkers, Lab Vision Corporation, Fremont, CA, USA	1:100	Mouse	Monoclonal	30 minutes	#MS-699-P	Ab-1/8G7G3/1
MMP14	Chemicon International, Millipore, Billerica, MA, USA	1:200	Mouse	Monoclonal	60 minutes	#MAB3329	
MMP-2	Neomarkers, Lab Vision Corporation, Fremont, CA, USA	1:100	Mouse	Monoclonal	60 minutes	#MAB13431	clone A-Gel VC2
RAR-β	Santa Cruz Biotechnology Inc., San Francisco, CA, USA	1:200	Rabbit	Polyclonal	60 minutes	#sc-56864	
SOX2	Chemicon International, Millipore, Billerica, MA, USA	1:500	Rabbit	Polyclonal	45 minutes	#AB5603	
