# Supplementary material for: Histological, immunohistochemical and transcriptomic characterization of human tracheoesophageal fistulas
Source: PLoS One. 2020 Nov 17;15(11):e0242167. doi: 10.1371/journal.pone.0242167 (PMC7671559; doi:10.1371/journal.pone.0242167)
Supplement: S10 File — (PDF) [file pone.0242167.s010.pdf]

S10 File: Immunostainings MMP2, MMP14 and BMPR1A

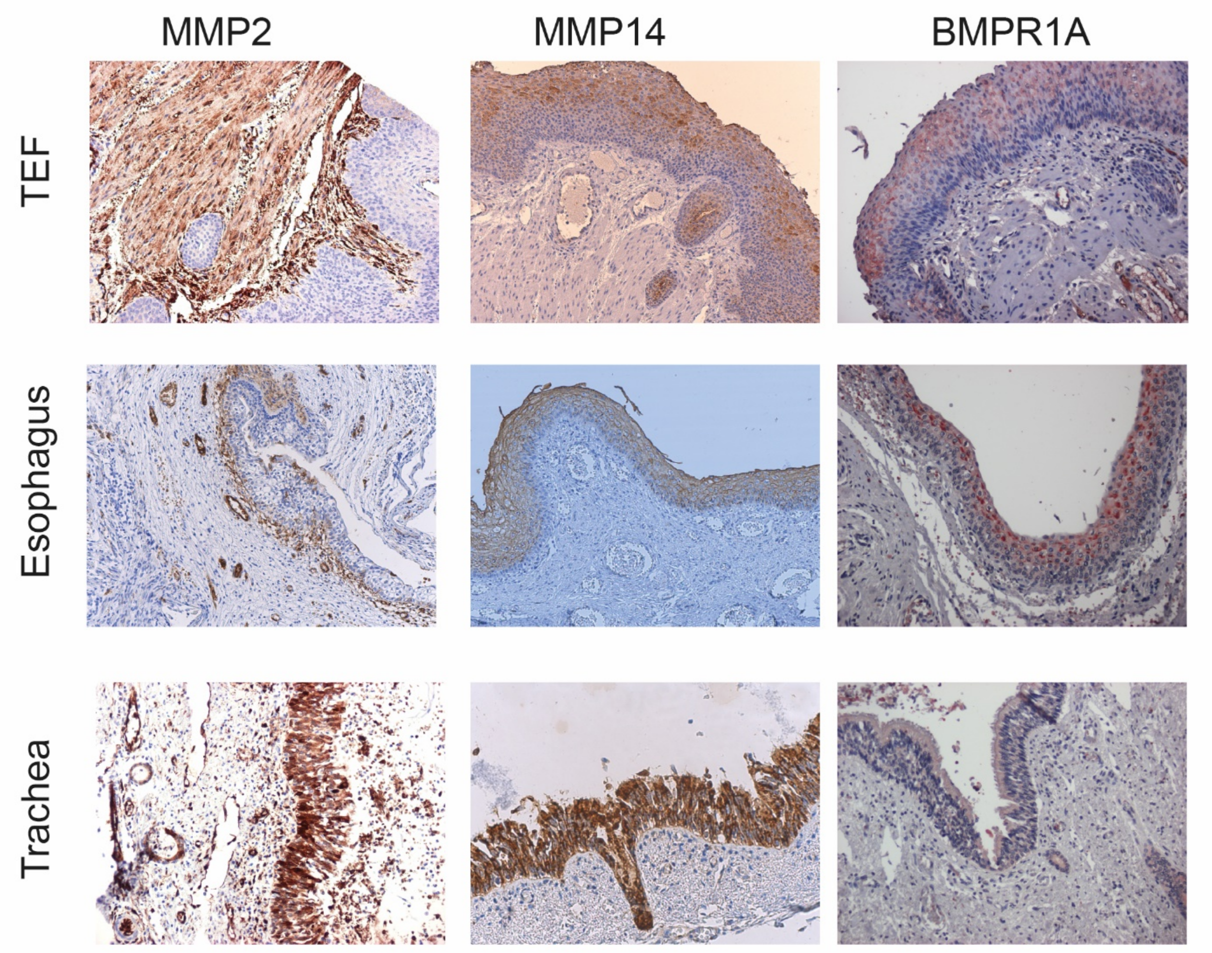

Immunohistochemical stainings of esophagus, trachea and TEF. All stainings at 20x magnification, except MMP14 esophagus.
