# Supplementary material for: Histological, immunohistochemical and transcriptomic characterization of human tracheoesophageal fistulas
Source: PLoS One. 2020 Nov 17;15(11):e0242167. doi: 10.1371/journal.pone.0242167 (PMC7671559; doi:10.1371/journal.pone.0242167)
Supplement: S11 File — (PDF) [file pone.0242167.s011.pdf]

S11 File: Causal network analysis using IPA©

We used the algorithm embedded in the Ingenuity Pathway Analysis tool to infer enriched pathways. Network significance scores (numerical value used to rank networks according to their degree of relevance to the dataset) were calculated. The score takes into account: the number of Network Eligible molecules in the network and its size, the total number of Network Eligible molecules analyzed and the total number of molecules in Ingenuity's knowledge base that could be included in networks. The score is the negative logarithm of the p-value calculated using the right-tailed Fisher's Exact test[1]. As thresholds for significance we used a P value ≤ 0.05 and a Z-score of 1.5 without correcting for multiple testing. Pathways are ranked according to their P-value and direction change of the pathway (Z-score). Pathways are derived by uploading the most significant (0.01 FDR) differential expressed genes from both the pairwise analysis of TEF vs Esophagus and TEF vs Trachea. We excluded the genes exclusively differentially expressed between Esophagus and Trachea. Visualization of these pathways is done using less stringent cut-offs in the differential expression analysis (parametric p-value of 0.05).

References

1. Kramer A, Green J, Pollard J, Jr., Tugendreich S. Causal analysis approaches in Ingenuity Pathway Analysis. Bioinformatics (Oxford, England). 2014;30(4):523-30. Epub 2013/12/18. doi: 10.1093/bioinformatics/btt703. PubMed PMID: 24336805; PubMed Central PMCID: PMCPMC3928520.
