# Supplementary material for: Histological, immunohistochemical and transcriptomic characterization of human tracheoesophageal fistulas
Source: PLoS One. 2020 Nov 17;15(11):e0242167. doi: 10.1371/journal.pone.0242167 (PMC7671559; doi:10.1371/journal.pone.0242167)
Supplement: S12 File — (PDF) [file pone.0242167.s012.pdf]

## S12 File: TEF specific expression pattern analysis

We determined which genes were not expressed in any of the control samples used, but were expressed in at least 11 TEFs. Moreover, we also determined which genes were not expressed in at least 11 TEF samples, but were expressed in all control samples. At the start of the analysis, all probe sets were characterized per sample to be “present”, “marginal” or “absent”, using information from Affymetrix GeneChip Operating Software. For this analysis, the “marginal” probe sets were left out. We tested for GO-term enrichment (GO Ontology database Released 2019-12-09) using the PANTHER Overrepresentation Test (Released 20190711) and all Homo sapiens genes as a reference. Annotation datasets used were Go biological process, molecular function and Cellular component. Statistical evaluation using Fisher exact and False discovery rate correction for multiple testing (FDR<0.05).
