# Supplementary material for: Histological, immunohistochemical and transcriptomic characterization of human tracheoesophageal fistulas
Source: PLoS One. 2020 Nov 17;15(11):e0242167. doi: 10.1371/journal.pone.0242167 (PMC7671559; doi:10.1371/journal.pone.0242167)
Supplement: S13 File — (PDF) [file pone.0242167.s013.pdf]

S13 File: Overlap with mouse developmental transcriptome

Using publicly available mouse developmental gene expression datasets, we determined which of the genes that were differentially expressed between TEF, esophagus and trachea were (differentially) expressed during early foregut development. We compared early foregut formation (E.8.25 endoderm, ectoderm and mesoderm), E11.5 esophagus [1] to controls (GSE13040) and evaluated the overlap with those in dissected mouse foreguts before (E8.5), during (E9.0) and when (E9.5) lung buds are formed in [2] (GSE19873). These datasets were downloaded from the gene expression omnibus [3] and imported into BRB-ArrayTools Version: 4.5.0 - Beta_2. (http://linus.nci.nih.gov/BRB-ArrayTools.html), annotated by Bioconductor (www.bioconductor.org), R version 3.2.2 Patched (2015-09-12 r69372) and normalized. Replicate spots within an array were averaged. Probes showing minimal variation across the set of arrays were excluded from the analysis. probes of which less than 10% of expression data had at least a 1.5 -fold change from the median were excluded as well as those in which 50% of arrays had missing data. The minimum fold change for the class comparisons was set at 2. We used a two-sample T-test with random variance model, permutation p-values for significant genes based on 10.000 random permutations and a nominal significance level of each univariate test of 0.05. Pathways are derived by uploading the most significant (0.01 FDR) differential expressed genes from both the pairwise analysis of TEF vs Esophagus and TEF vs Trachea into IPA©.

References

1. Sherwood RI, Chen TY, Melton DA. Transcriptional dynamics of endodermal organ formation. Developmental dynamics : an official publication of the American Association of Anatomists. 2009;238(1):29-42. Epub 2008/12/20. doi: 10.1002/dvdy.21810. PubMed PMID: 19097184; PubMed Central PMCID: PMCPmc3756511.
2. Millien G, Beane J, Lenburg M, Tsao PN, Lu J, Spira A, et al. Characterization of the mid-foregut transcriptome identifies genes regulated during lung bud induction. Gene expression patterns : GEP. 2008;8(2):124-39. Epub 2007/11/21. doi: 10.1016/j.modgep.2007.09.003. PubMed PMID: 18023262; PubMed Central PMCID: PMCPmc2440337.
3. Edgar R, Domrachev M, Lash AE. Gene Expression Omnibus: NCBI gene expression and hybridization array data repository. Nucleic acids research. 2002;30(1):207-10. Epub 2001/12/26. PubMed PMID: 11752295; PubMed Central PMCID: PMCPmc99122.
