# Supplementary figures and images for: Histological, immunohistochemical and transcriptomic characterization of human tracheoesophageal fistulas
Source: PLoS One. 2020 Nov 17;15(11):e0242167. doi: 10.1371/journal.pone.0242167 (PMC7671559; doi:10.1371/journal.pone.0242167)

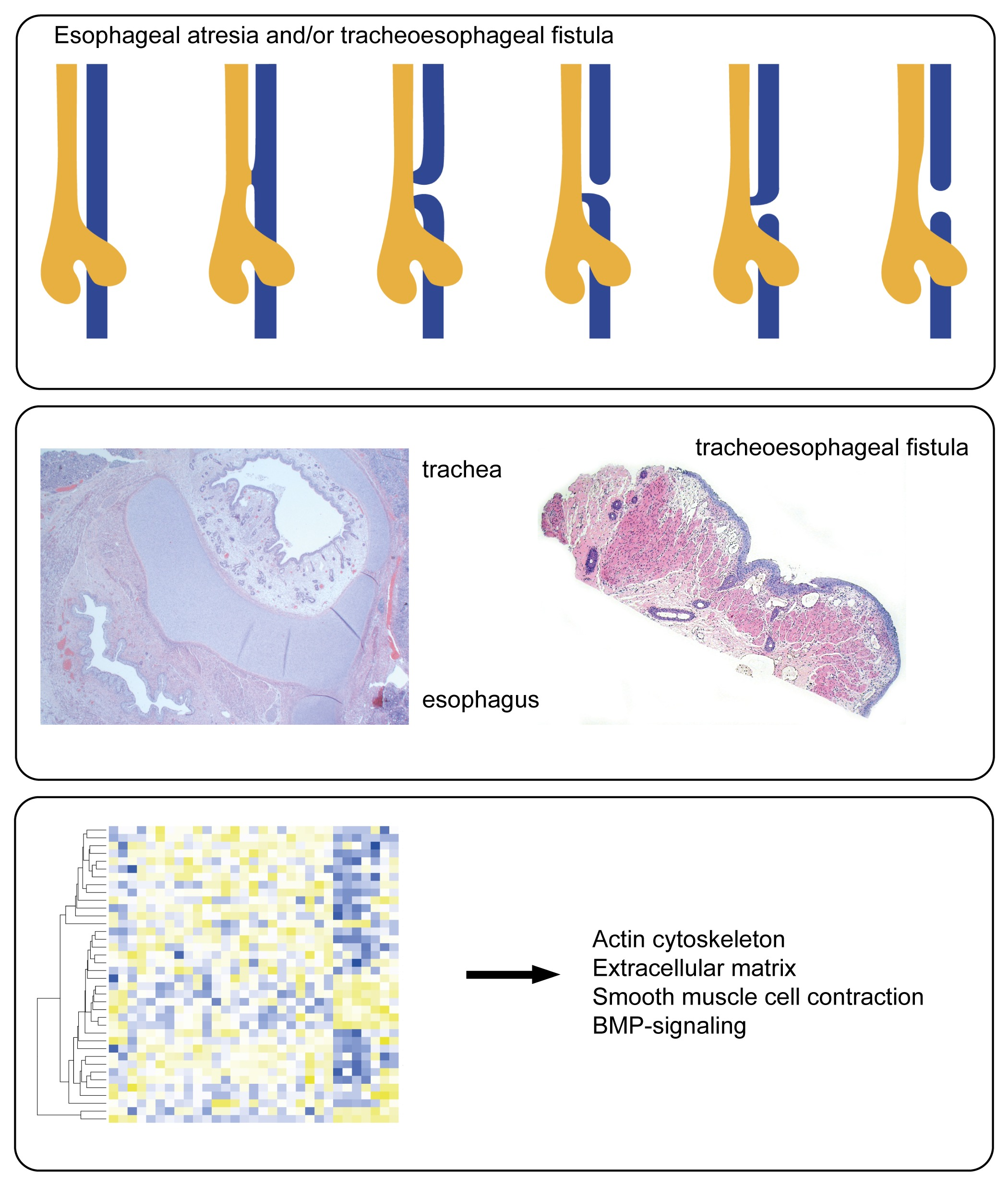

Supplement: S1 Graphical abstract — (TIF) [file pone.0242167.s014.tif]
